# On *Lactococcus lactis* UL719 competitivity and nisin (Nisaplin^®^) capacity to inhibit *Clostridium difficile* in a model of human colon

**DOI:** 10.3389/fmicb.2015.01020

**Published:** 2015-09-25

**Authors:** Christophe Le Lay, Benoit Fernandez, Riadh Hammami, Marc Ouellette, Ismail Fliss

**Affiliations:** ^1^STELA Dairy Research Center, Nutrition and Functional Foods Institute, Université Laval, QuébecQC, Canada; ^2^Centre de Recherche en Infectiologie de l’Université Laval, Axe Maladies Infectieuses et Immunitaires, Centre de Recherche du CHU de Québec, QuébecQC, Canada; ^3^Département de Microbiologie-Infectiologie et d’Immunologie, Faculté de Médecine, Université Laval, QuébecQC, Canada

**Keywords:** *Clostridium difficile*, probiotic, *Lactococcus lactis* UL719, bacteriocin, nisin, colon model

## Abstract

*Clostridium difficile* is the most frequently identified enteric pathogen in patients with nosocomially acquired, antibiotic-associated diarrhea and pseudomembranous colitis. Although metronidazole and vancomycin were effective, an increasing number of treatment failures and recurrence of *C. difficile* infection are being reported. Use of probiotics, particularly metabolically active lactic acid bacteria, was recently proposed as an alternative for the medical community. The aim of this study was to assess a probiotic candidate, nisin Z-producer *Lactococcus lactis* UL719, competitivity and nisin (Nisaplin^®^) capacity to inhibit *C. difficile* in a model of human colon. Bacterial populations was enumerated by qPCR coupled to PMA treatment. *L. lactis* UL719 was able to survive and proliferate under simulated human colon, did not alter microbiota composition, but failed to inhibit *C. difficile*. While a single dose of 19 μmol/L (5× the MIC) was not sufficient to inhibit *C. difficile*, nisin at 76 μmol/L (20×the MIC) was effective at killing the pathogen. Nisin (at 76 μmol/L) caused some temporary changes in the microbiota with Gram-positive bacteria being the mostly affected. These results highlight the capacity of *L. lactis* UL719 to survive under simulated human colon and the efficacy of nisin as an alternative in the treatment of *C. difficile* infections.

## Introduction

*Clostridium difficile* is a Gram-positive anaerobic sporulating pathogen causing intestinal infections following disturbance of the human and animal gut microbiota, usually subsequent to an antibiotic therapy. *C. difficile* is now thought to be responsible for a wide range of diseases including acute diarrhea and pseudomembranous colitis, and could lead to colonic perforation and death if untreated (Borriello et al., 1990). Although metronidazole and vancomycin are well-established treatments for *C. difficile* infections (CDI) (Surowiec et al., 2006; Kelly and LaMont, 2008), an increasing number of treatment failures with these antibiotics and recurrence of *C. difficile* infection are being reported, reviewed in Vardakas et al. (2012). Vancomycin is also losing its attractiveness for CDI treatment with emergence of vancomycin-resistant enterococci and dissemination of antibiotic-resistance determinants within the hospital environment (Lagrotteria et al., 2006). The emergence of *C. difficile* isolates with multiple-drug resistance is rarely explicitly mentioned (Peláez et al., 2002; Mutlu et al., 2007), but constitutes further a serious public health threat that urges the need of novel antimicrobial treatments.

Previously, a large number of clinical trials highlighted the positive role of probiotics in the treatment of diarrhea by either shortening its duration and/or preventing its complications in infants and young children, reviewed in Guandalini (2011). In instance, a yogurt containing a combination of *Lactobacillus rhamnosus* GG, *L. acidophilus* La-5, and *Bifidobacterium lactis* Bb12 was shown to be an effective method for reducing the incidence of antibiotic-associated diarrhea in children (Fox et al., 2015). Moreover, different probiotics (*Saccharomyces boulardii, L. casei* DN114001, a mixture of *L. acidophilus* and *B. bifidum*, and a mixture of *L. acidophilus, L. casei* and *L. rhamnosus*) significantly improved CDI prevention, reviewed in McFarland (2015). Although several meta-analyses pointed the positive effect of probiotics, their role in the prevention of CDI remains unclear. The health-promoting properties of probiotics are numerous and their effects on host include competition with pathogens for adhesion sites and nutrients, stimulation of immunity/immunomodulation, and production of inhibitory substances such as bacteriocins (Fliss et al., 2011). Bacteriocins have been suggested as promising alternative to conventional antibiotics (Rea et al., 2007, 2010), and their production is being considered as a probiotic trait although not clearly demonstrated *in vivo* (Dobson et al., 2012). While several bacteriocins including nisin (Le Blay et al., 2007; Le Lay et al., under revision), Microbisporicin (Castiglione et al., 2008), Lacticin 3147 (Rea et al., 2007) and thuricin CD (Rea et al., 2010) were shown effective against *C. difficile*, to date only nisin is approved by the American Food and Drug Administration, the World Health Organization, and the European Union as natural food additive (Delves-Broughton, 1990). Nisin displays high antibacterial activity against multi-resistant *Streptococcus pneumoniae*, methicillin-resistant *Staphylococcus aureus* (MRSA), vancomycin-resistant *Enterococcus faecium, E. faecalis*, and *C. difficile* (Severina et al., 1998; Le Blay et al., 2007).

Previously, we have observed that potential probiotic *Lactococcus lactis* UL719, a nisin Z producer, was able to survive through the gastrointestinal tract (unpublished data). The strain *L. lactis* UL719 was able to grow and inhibit *Listeria* in a medium simulating the nutrient composition of the human colon (Fernandez et al., 2013). The aim of this study was to evaluate the capacity of *L. lactis* UL719 and nisin (Nisaplin^®^) to inhibit *C. difficile* in a model of the colon mimicking physiological and microbiological conditions of the large intestine. In addition, impact of both strain and its bacteriocin on the gut microbiota composition were also investigated.

## Materials and Methods

### Bacterial Strains and Growth Conditions

*Lactococcus lactis* sp. *lactis* biovar. *diacetylactis* UL719, a nisin Z-producer, was isolated from raw milk cheese (Ali et al., 1995; Meghrous et al., 1997). *C. difficile* ATCC43255 was purchased from ATCC (American Type Culture Collection, Manassas, VA, USA). *L. lactis* UL719 was reactivated in De Man Rogosa Sharpe (MRS) broth (Difco Laboratories, Sparks, MD, USA) and cultivated for 24 h at 30°C. *C. difficile* was reactivated in Brain Heart Infusion (BHI) broth (Difco laboratories, Sparks, MD, USA) supplemented with 0.05% L-cysteine-HCl (Sigma chemicals). *C. difficile* culture was cultivated in an anaerobic chamber (Forma scientific anaerobic system Model 1025; Forma Scientific, Marietta, OH, USA) at 37°C for 24 h. *Escherichia coli* ATCC25922 and *E. faecalis* ATCC27275 were grown aerobically at 37°C for 24 h in BHI and TSBYE, respectively. *B. adolescentis* ATCC15703, *Bacteroides thetaiotaomicron* ATCC29741, *Blautia coccoides* ATCC29236, and *C. leptum* ATCC29065 were, respectively, grown in MRS broth (0.05% L-cysteine-HCl), BHI (0.05% L-cysteine-HCl) and a modified chopped meat medium with maltose (ATCC medium 2751) under anaerobic conditions at 37°C. All bacterial strains were maintained in 20% glycerol at -80°C. Prior to each experiment, each bacterial strain was subcultured at least three times (inoculation at 1%, v/v) at 24 h intervals.

### Development of Large Intestine Fermentation Model

#### Feces Collection and Immobilization in Gel Beads

A fresh fecal sample was obtained from one 27 years old healthy donor who had not taken antibiotics for the previous 3 months. The collected fecal sample was used for immobilization following procedure described by Le Blay et al. (2012). The entire process was completed aseptically under anaerobic conditions within 1 h after sample collection.

### Nutritive Medium

The culture medium used for colonic fermentation was the same as described by Macfarlane et al. (1998) with some modifications. Briefly, 0.5 mL of a vitamin solution (mg/L: pyridoxine–HCl 20; p-aminobenzoic acid 10; nicotinic acid 10; biotin 4; folic acid 4; vitamin B12 1; thiamine 8; riboflavin 10; menadione 2; vitamin K1 0.005; pantothenate 20) described by Gibson and Wang (1994) was added to each liter of the culture medium. The vitamin solution was sterilized by filtration (0.2 μm, VWR) and added to the autoclaved medium (15 min, 121°C) after cooling at room temperature.

### Experimental Setup and Sampling

The colonic fermentation was based on the model described by Cinquin et al. (2004). A single-stage reactor (Bioflo III, New Brunswick Scientific Inc., Edison, NJ, USA) with 1 L working volume containing 30% (v/v) of freshly prepared beads was used to mimic the microbial ecosystem of adult distal colon. The colonization of beads with fecal microbiota was carried out during 2 days, and the nutritive medium was aseptically replaced by fresh culture medium every 12 h. pH (6.2) and anaerobic and temperature (37°C) conditions were maintained during the whole fermentation by addition of 5 M NaOH and a continuous flow of pure CO_2_ in the headspace. The continuous fermentation was carried out in the same reactor connected to a stirred feedstock vessel containing the sterile culture medium at 4°C under a CO_2_ atmosphere and to an eﬄuent-receiving vessel. Feed flow rate was adjusted to 83.3 mL/h to mimic a mean retention time of 12 h encountered in adult distal colon.

**FIGURE 1:**
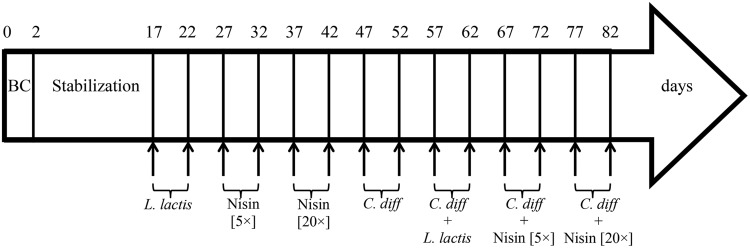
**Time schedule of continuous intestinal fermentation during the different treatment periods.** BC, bead colonization. *Lactococcus lactis* UL719 was added at final concentration of 10^9^ CFU/mL in the reactor. *Clostridium difficile* ATCC43255 was added at a final concentration of 5 × 10^6^ CFU/mL.

The fermentation process was carried out for a total of 82 days and microbiota was stabilized 2 weeks before challenging tests. First, a cell suspension of *L. lactis* UL719 (at final concentration 10^9^ CFU/mL in the reactor) was added twice to the reactor (day 17 and 22) (**Figure 1**). Then, nisin A (Nisaplin^®^, Danisco, Copenhagen, Denmark) was added to the reactor at 5× (at day 27 and 32) and 20× (at day 37 and 42) the MIC (3.8 μmol/L vs. *C. difficile*) to measure the impact of high doses of nisin on the intestinal flora. Next, challenges with *C. difficile* ATCC43255 (at a final concentration of 5 × 10^6^ CFU/mL in the reactor) in absence (day 47 and 52) or in presence of *L. lactis* (added at a final concentration of 10^9^ CFU/mL in the reactor; day 57 and 62) or in presence of different concentrations of nisin A [5× (day 67 and 72) or 20× (day 77 and 82) the MIC] were performed. Samples were collected for bacterial enumeration by qPCR. After each addition, samples were hourly taken during first 4 and at 8 h.

### Microbiota Composition Analysis using q-PCR Coupled to PMA Treatment

Standard curve for the qPCR quantification was done using the following strains: *E. coli* ATCC 25922, *B. adolescentis* ATCC15703, *B. thetaiotaomicron* ATCC29741, *C. leptum* ATCC29065, *B. coccoides* ATCC29236, and *E. faecalis* ATCC27275. Samples were collected from the reactor and treated with propidium monoazide (Biotium, Inc., Hayward, CA, USA) prior enumeration of viable bacteria, as described in Fernandez et al. (2015). The DNA from fecal and fermentation samples were then extracted following the protocol of Ahlroos and Tynkkynen (2009) using the Wizard^®^genomic DNA Purification Kit (Promega, Madison, WI, USA) with some modifications (Fernandez et al., 2015). Real-time PCR was carried out on an ABI 7500 real-time PCR system (Applied biosystem, Streetsville, ON, Canada) with the iTaq^TM^ Universal SYBR^®^Green supermix (Biorad, Oakville, ON, Canada) in 96-well plates following method described in Fernandez et al. (2015). Primers used in this study are summarized in **Table 1**. Each sample was done in triplicate.

**Table 1:** Primers used for the detection of different bacterial groups in inoculum or fermentation samples by real-time qPCR analysis.

Target organisms	Gene	Sequence 5′–3′	Reference
All bacteria	*16S*	f: TCCTACGGGAGGCAGCAGT	Hopkins et al., 2005
		r: GGACTACCAGGGTATCTAATCCTGTT	
*Ruminococcaceae (Clostridium leptum)*	*16S*	f: GCACAAGCAGTGGAGT	Kanno et al., 2009
		r: CTTCCTCCGTTTTGTCAA	
*Lachnospiraceae (Blautia coccoides)*	*16S*	f: AAATCACGGTACCTGACTAA	Matsuki et al., 2002
		r: CTTTGAGTTCATTCTTGCGAA	
*Bifidobacteria* sp.	*16S*	f: TCGCGTC(C/T)GGTGTGAAAG	Rinttilä et al., 2004
		r: CCACATCCAGC(A/G)TCCAC	
*Enterobacteriaceace* sp.	*16S*	f: CATGCCGCGTGTATGAAGAA	Huijsdens et al., 2002
		r: CGGGTAACGTCAATGAGCAAA	
*Bacteroidetes*	*16S*	f: GGTGTCGGCTTAAGTGCCAT	Rinttilä et al., 2004
		r: CGGA(C/T)GTAAGGGCCGTGC	
*Lactobacillaceae/Leuconostocaceae* group	16S	f: AGCAGTAGGGAATCTTCCA	Rinttilä et al., 2004
		r: CGCCACTGGTGTTCYTCCATATA	
*C. difficile*	*tcdA*	f: CTCCTGGACCACTTAAACTTATTGTG	This study
		r: GCTACCGCAGAAAACTCTATGTTTT	
*Lactococcus lactis* UL719	*nisI*	f: CTGAAGTACGTGTGTTTGATTCAGTTAG	Trmčiać et al., 2011
		r: TCGCCATAATCCCATTCCGTC	

### Analyses of Metabolites

Short-chain fatty acids (SCFA: acetate, propionate, butyrate, and valerate) and isoacids (isobutyrate and isovalerate) were determined by high-performance liquid chromatography (HPLC) analysis (Waters, Milford, MA, USA) equipped with an Ion 300 column (Transgenomic, San Jose, CA, USA), a differential refractometer (Model R410, Waters) as previously described by Cinquin et al. (2004). The analysis was performed at a flow rate of 0.4 mL/min at 37°C, with an injection volume of 100 μL. Each analysis was done in duplicate. The mean metabolite concentrations were expressed in mmol/L.

### Statistical Analysis

Data are presented as means ± SD. Cell counts values were log_10_-transformed and analyzed for repeated measures using the PROC MIXED procedure of SAS v9.2 statistical package (SAS Institute Inc., Cary, NC, USA). The statistical differences in metabolites contents between treatments were evaluated using a one-way ANOVA *t*-test. The level of significance was *P* ≤ 0.05.

## Results

### Microbiota Composition during Stabilization Period

Bacterial populations enumerated by qPCR coupled to PMA treatment in the fecal inoculum and eﬄuent samples at the end of stabilization period are summarized in **Table 2**. The fecal inoculum presented a total bacterial cell counts of 11.84 ± 0.04 log_10_ CFU/g, which was dominated by *Bacteroidetes* (10.85 ± 0.02 log_10_ CFU/g), clostridia (10.55 ± 0.02 log_10_ CFU/g), and bifidobacteria (10.16 ± 0.15 log_10_ CFU/g). At the end of the stabilization period (16 days) under simulated colon conditions, the microbiota population reached a pseudo steady state in which a slight change was observed in the microbial balance, compared to the initial fecal inoculum. The microbiota decreased by -0.7 log_10_ CFU/mL at this stage and was dominated by *Bacteroidetes* group with 10.52 ± 0.08 log_10_ CFU/mL. While *Enterobacteriaceae* group increased by 1.49 log_10_ and reached 8.73 ± 0.01 log_10_ CFU/mL, bifidobacteria, and *Lactobacillaceae/Leuconostocaceae* group populations dropped to 6.14 ± 0.08 and 3.82 ± 0.14 log_10_ CFU/mL, respectively. Nevertheless, the obtained results are in accordance to those previously reported for colonic fermentation models (Brück et al., 2002; Probert and Gibson, 2004; Cleusix et al., 2008; Le Blay et al., 2012).

**Table 2:** Bacterial cell counts in the fecal inoculum and during the fermentation at the end of the stabilization period of the continuous culture measured by qPCR.

Bacterial population	Feces^a^	Inoculum^b^	End of stabilization^c^	CFS^d^
		qPCR	qPCR	
Total bacteria	10.6–11.6	10.84 ± 0.04	11.11 ± 0.17	8.9–10.2
*Lachnospiraceae* group	9.9–11.1	9.46 ± 0.02	8.14 ± 0.05	7–9.5
*Ruminococcaceae* group		10.52 ± 0.02	9.43 ± 0.01	
*Bacteroidetes*	9.2–10.3	10.85 ± 0.02	10.52 ± 0.08	6.8–10.1
*Bifidobacterium* sp.	7.2–10.2	10.16 ± 0.15	6.14 ± 0.08	6.0–9.0
*Enterobacteriaceae* sp.	8.0–9.8	7.24 ± 0.53	8.73 ± 0.01	7.3–9.2
*Lactobacillaceae/Leuconostocaceae* group	8.6–9.5	6.98 ± 0.19	3.82 ± 0.14	<6.0–8.3

*^a^Fecal populations (log_10_ CFU/g) of healthy adults reported in various studies and measured by FISH (Franks et al., 1998; Harmsen et al., 1999).*

*^b^Bacterial concentrations (log_10_ CFU/g of feces).*

*^c^Bacterial concentrations (log_10_ CFU/mL).*

*^d^Bacterial concentrations (log_10_ CFU/mL) reported for *in vitro* continuous fermentation samples (CFS) and measured by FISH (Brück et al., 2002; Probert and Gibson, 2004; Cleusix et al., 2008; Le Blay et al., 2012).*

### *Lactococcus lactis* UL719 Alone or in Presence of *C. difficile* ATCC43255 have no Perturbing Impact on Intestinal Microbiota under Simulated Colonic Conditions

**FIGURE 2:**
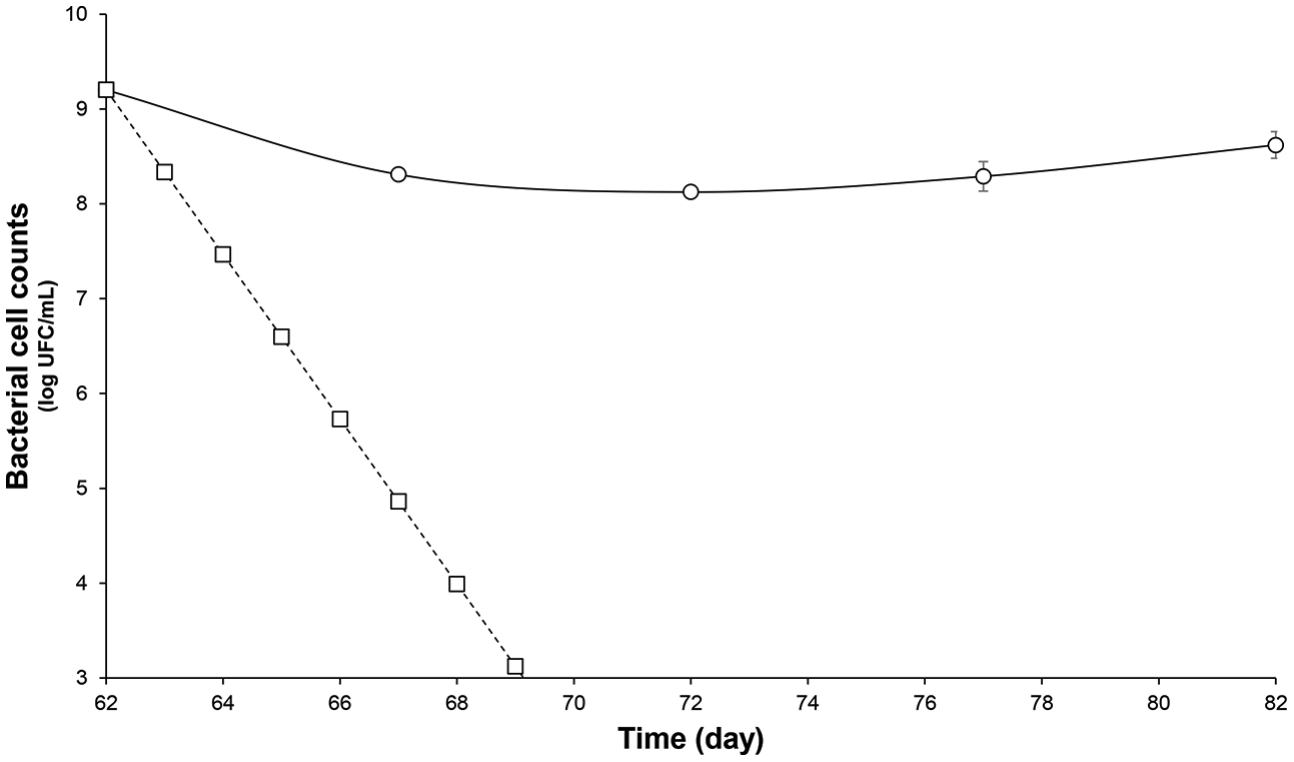
**Survival of *L. lactis* UL719 after its last addition (day 62) in a human colon model.** *L. lactis* UL719 (circle); theoretical washout (square).

**FIGURE 3:**
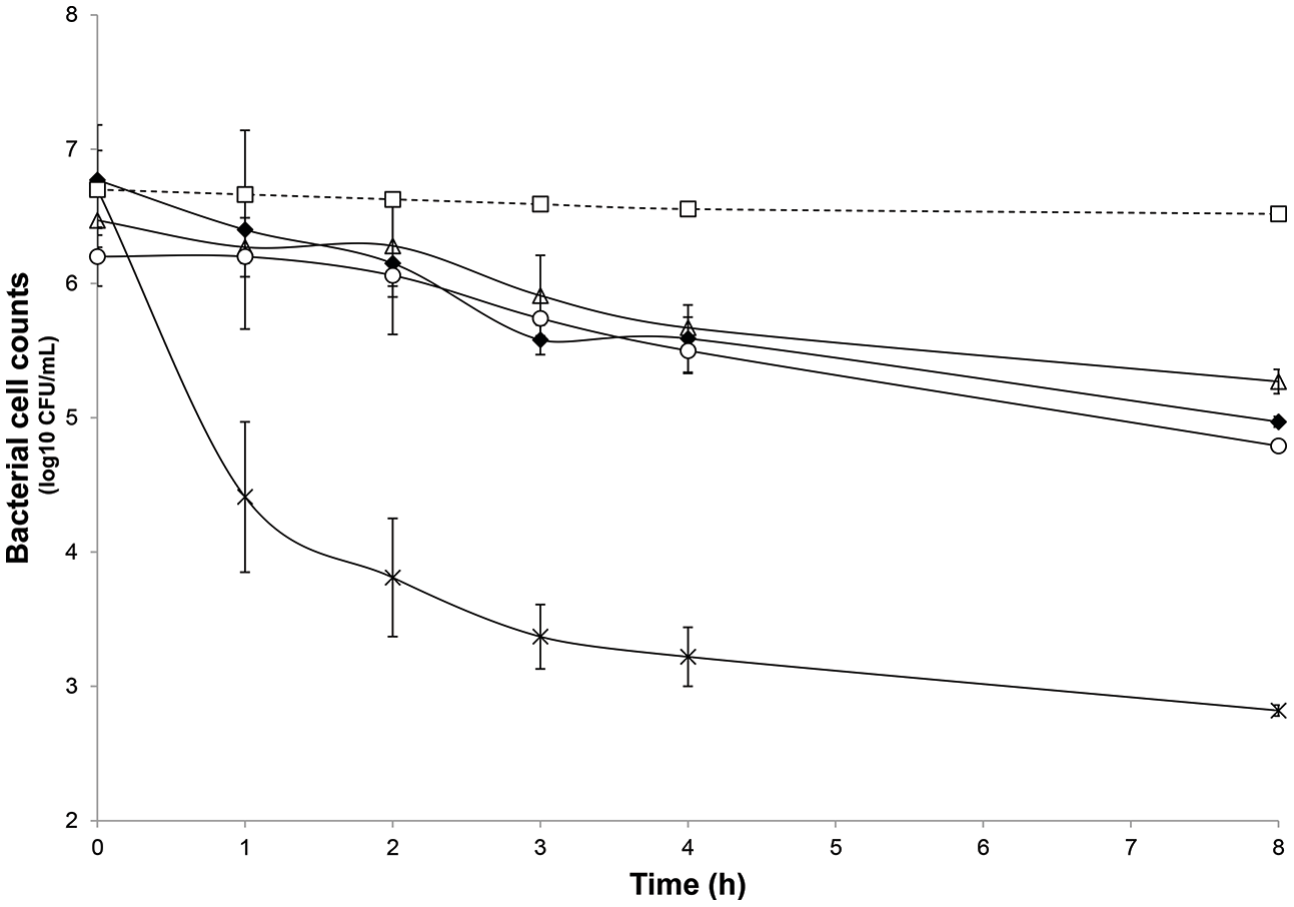
**Inhibitory activity of nisin at 5× and 20× the MIC (3.8 μmol/L) and *L. lactis* UL719 (10^9^ CFU/mL) against *C. difficile* ATCC43255 in a human colon model.** *C. difficile* alone (black diamond); *C. difficile* plus nisin 5× (white triangle); *C. difficile* plus nisin 20× (cross); *C. difficile* plus *L. lactis* UL719 (white circle); theoretical washout (white square).

After the stabilization period, *L. lactis* UL719, *C. difficile* ATCC43255, and their combination were successively added to the bioreactor and the microbiota populations were monitored by qPCR (**Table 3**). Interestingly, the addition of *L. lactis* UL719 at 1 × 10^9^ CFU/mL to the bioreactor, did not induce any significant change neither in the intestinal microbiota composition nor in metabolites production (**Table 4**). Since the last addition of *L. lactis* UL719 to the reactor, the strain was detected at about 0.1 - 1 × 10^9^ CFU/mL during the remaining 20 days of fermentation (**Figure 2**). While the infection of the bioreactor with 5 × 10^6^ CFU/mL of *C. difficile* did not affect the microbiota composition, a slight but significant decrease (*p* < 0.05) of acetate and butyrate was detected (from 76.24 to 72.59 mmol/L and from 32.13 to 29.54 mmol/L, respectively) (**Table 4**). Simultaneous addition of *C. difficile* and *L. lactis* UL719 had no impact on the microbiota cell counts but a significant decrease (*p* < 0.05) of butyrate (from 32.13 to 28.40 mmol/L). Under these conditions, *L. lactis* has no inhibitory effect on *C. difficile* (**Figure 3**).

**Table 3:** Impact of *L. lactis* UL719 (10^9^ CFU/mL) and/or *C. difficile* ATCC43255 (5 × 10^6^ CFU/mL) addition on the microbiota.

Bacterial population	*L. lactis* UL719>		*C. difficile*		*L. lactis* UL719 + *C. difficile*	
			
	0	8 h	0	8 h	0	8 h
Total bacteria	10.87 ± 0.13	10.80 ± 0.19	10.80 ± 0.08	10.71 ± 0.04	10.40 ± 0.31	10.66 ± 0.19
*Lachnospiraceae* group	8.05 ± 0.25	8.04 ± 0.28	8.06 ± 0.09	8.01 ± 0.05	. 7.42 ± 0.24	7.76 ± 0.23
*Ruminococcaceae* group	9.36 ± 0.10	9.32 ± 0.17	9.29 ± 0.03	9.25 ± 0.02	8.93 ± 0.21	9.23 ± 0.09
*Bacteroidetes*	10.51 ± 0.04	10.32 ± 0.11	10.31 ± 0.16	10.06 ± 0.13	9.82 ± 0.60	10.18 ± 0.33
Bifidobacteria	6.35 ± 0.17	6.13 ± 0.79	5.26 ± 0.84	5.18 ± 0.81	5.56 ± 0.69	5.61 ± 0.07
*Enterobacteriaceae*	8.95 ± 0.10	8.81 ± 0.22	9.49 ± 0.08	9.28 ± 0.04	9.20 ± 0.31	9.23 ± 0.25
*Lactobacillaceae/Leuconostocaceae* group	3.69 ± 0.15	3.57 ± 0.47	5.75 ± 0.44	5.46 ± 0.36	5.43 ± 0.28	5.59 ± 0.32

*Data are mean ± standard deviation of two biological replicates and three technical replicates. Values are not significantly different by the student’s test (*P* < 0.05).*

**Table 4:** Concentration of short chain fatty acids (SFCA) in eﬄuent samples at 4 h following various treatments.

	Treatments											
Metabolites	End of stabilization		*L. lactis* UL719		*C. difficile*		*L. lactis* UL719 + *C. difficile*		Nisin 5×		Nisin 20×	
						
	(mmol/L)	(%)	(mmol/L)	(%)	(mmol/L)	(%)	(mmol/L)	(%)	(mmol/L)	(%)	(mmol/L)	(%)
Acetate	76.24 ± 0.15^a^	44.67	75.34 ± 1.23^a^	43.94	72.59 ± 1.73^b^	44.62	76.30 ± 0.45^a^	45.18	75.14 ± 0.67^a^	44.47	69.12 ± 2.44^c^	43.86
Propionate	43.09 ± 0.01^c^	25.25	43.29 ± 0.26^c^	25.25	41.68 ± 2.27^c^	25.62	43.53 ± 2.42^c^	25.78	45.54 ± 1.51^b^	26.95	48.69 ± 0.33^a^	30.89
Butyrate	32.13 ± 0.01^a^	18.82	32.59 ± 0.26^a^	19.01	29.54 ± 0.70^c^	18.16	28.40 ± 0.44^c^	16.82	30.83 ± 0.30^b^	18.25	26.29 ± 0.75^d^	16.68
Isobutyrate	5.23 ± 0.47^b^	3.06	5.75 ± 0.24^a^	3.35	5.44 ± 0.30^ab^	3.334	5.61 ± 0.08^ab^	3.32	4.47 ± 0.42^c^	2.65	4.44 ± 0.19^c^	2.82
Valerate	5.00 ± 0.08^a^	2.93	4.92 ± 0.24^a^	2.87	4.64 ± 0.68^a^	2.85	5.89 ± 0.15^a^	3.49	4.84 ± 0.59^a^	2.86	2.05 ± 2.37^b^	1.30
Isovalerate	8.99 ± 0.39^b^	5.27	9.56 ± 0.18^a^	5.58	8.81 ± 0.31^b^	5.41	9.13 ± 0.15^ab^	5.41	8.15 ± 0.49^c^	4.82	7.02 ± 0.30^d^	4.45
*Total SCFA*	170.68		171.45		162.70		168.86		168.97		157.61	

*Data are mean ± standard deviation of two biological replicates and three technical replicates. Values with different letters in the same row are significantly different by the student’s test (*P* < 0.05).*

### A Nisin Concentration of 20× the MIC is Required to Effective Inhibition of *C. difficile* ATCC43255 in a Model of Human Colon

**FIGURE 4:**
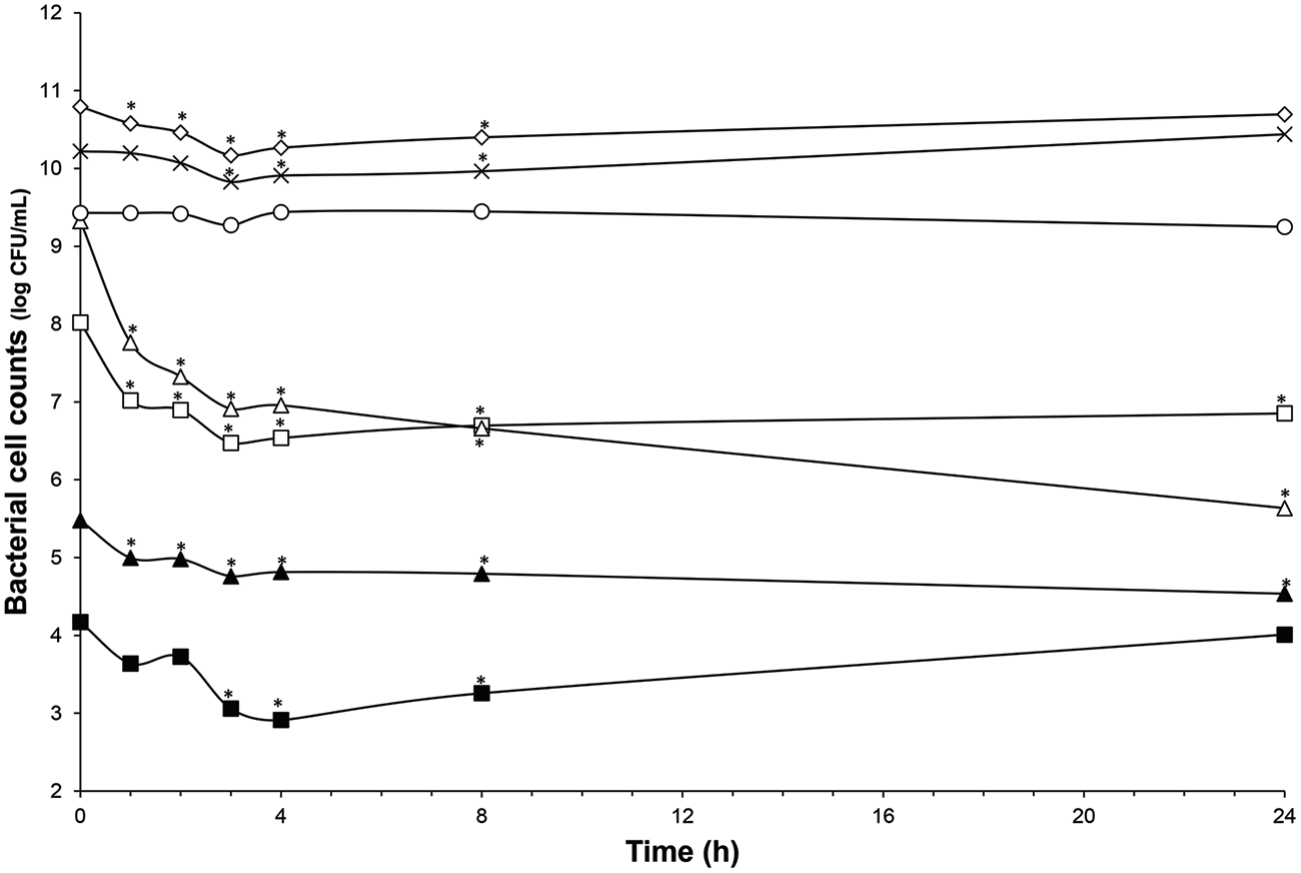
**Impact of nisin addition at 76 μmol/L (20× the MIC vs. *C. difficile* ATCC43255) on microbiota population enumerated by qPCR.** Total bacteria (white diamond); *Lachnospiraceae* group. (white square); *Ruminococcaceae* group. (white triangle); *Bacteroidetes* (cross); Bifidobacteria (black triangle); *Enterobacteriaceae* (white circle); *Lactobacillaceae/Leuconostocaceae* group. (black square); Values with asterisk are significantly different (*P* < 0.05).

The microbiota was challenged by 5× and 20× the MIC vs. *C. difficile* ATCC43255. Nisin at 5× the MIC did not alter the microbiota which remained stable (data not shown) although minor variations in the metabolite production profile (**Table 4**). At a nisin concentration of 20× the MIC, total microbiota significantly decreased by 0.7 log_10_ (*p* < 0.008), as shown in **Figure 4**. Gram-positive bacteria were affected by this higher amount of nisin, with *Ruminococcaceae* group being the mostly altered (-3.7 log_10_) after 24 h. In a lesser extent, a reduction of 1.5 log_10_, 1.3 log_10_, and 1 log_10_ were recorded for *Lachnospiraceae* group, *Lactobacillaceae/Leuconostocaceae* group and bifidobacteria, respectively. After 24 h of nisin administration, all bacterial populations recovered their initial counts except *Ruminococcaceae* group which dropped to its minimum counts. While acetate and butyrate significantly decreased (*p* < 0.05) from 76.24 and 32.13 mmol/L to 69.12 and 26.29 mmol/L, propionate production increased by 13% (**Table 4**). Besides, a nisin concentration of 5× did not inhibit *C. difficile*, which counts remained close to control (*C. difficile* alone) (**Figure 3**). Conversely, nisin at 20× was effective at inhibiting *C. difficile* with a significant reduction (*p* < 0.001) of 2.3 log_10_ at 1 h that lasted for 8 h (**Figure 3**). *C. difficile* was not detected after 24 h in this model (data not shown).

## Discussion

Previously, we have demonstrated the nisin efficacy against several clinical isolates of *C. difficile* vegetative cells and spores (Le Lay et al., under revision). In addition, we have observed that *L. lactis* UL719, a nisin Z producer, was able to survive these GIT stressful conditions, to keep ability to produce its bacteriocin, and to reach the colon in large enough numbers (>10^8^ CFU) to comply with the recommended daily dose of 10^8^–10^9^ cells delivery to exert a beneficial effect on the host (unpublished data). The aim of this study was to assess *L. lactis* UL719 competitivity and nisin capacity to inhibit *C. difficile* ATCC43255 in a model of human colon. In this study, *L. lactis* UL719 at 10^9^ CFU/mL did not induce any significant change neither in the intestinal microbiota composition nor in metabolites production. The strain was monitored by quantification of *nisI* gene by PMA-qPCR, and found able to survive and proliferate up to 10^8^ CFU/mL in our colonic model during the 82 days of fermentation (**Figure 2**). Unlikely, *L. lactis* DPC6520 was shown more susceptible to GIT conditions, which cell counts were reduced by 10 000-fold 24 h after its inoculation into a colon model (Dobson et al., 2011). Likewise, a 19 μmol/L concentration of nisin (corresponding to 5× the MIC vs. *C. difficile* ATCC43255) did not alter microbiota levels. At a higher concentration of 76 μmol/L (20×), Gram-positive bacteria were affected and *Ruminococcaceae* group was the mostly altered (-3.7 log_10_), while increase in Gram-negative population (*Bacteroidetes* and *Enterobacteriaceae*) were observed. Nevertheless, the initial bacterial balance was quickly restored within 24 h after the addition of 20× nisin. Previously, we have shown *in vitro* the sensitivity of colonic Gram-positive bacteria such as *B. bifidum* DSM 20456, *L. fermentum* ETHZ, *C. clostridioforme* DSM933, *Eubacterium biforme* DSM3989 to nisin (Le Blay et al., 2007). Recently, Rea et al. (2011) reported that lacticin 3147 induce similar variations in microbiota composition, with a decrease in Firmicutes abundance in favor of *Proteobacteria*. Broad-spectrum antibiotics like vancomycin and metronidazole seems to induce also decrease of Firmicutes and an increase in *Enterobacteriaceae* and *Proteobacteria* (Antonopoulos et al., 2009; Rea et al., 2011). More recently, thuricin CD, a narrow spectrum bacteriocin produced by *Bacillus thuringiensis*, was used in the distal colon model and had no significant impact on the composition of the microbiota (Rea et al., 2011).

Although its capacity to survive colonic conditions, *L. lactis* UL719 had no significant effect on *C. difficile*. Similar results were previously reported with *L. lactis* DPC6520 (a lacticin 3147 producer) and *L. lactis* DPC6519 (lacticin non-producer) in an *ex vivo* human colonic model (Dobson et al., 2011). Although *L. lactis* UL719 is able to produce nisin in a Macfarlane medium simulating the nutrient composition of the colon (Fernandez et al., 2013), the lack of effectiveness observed here is likely due to no or a low production of nisin, not sufficient to inhibit *C. difficile*. Conversely, *L. salivarius* UCC118 has demonstrated its capacity to produce the Abp118 bacteriocin *in vivo* and to protect mice against infection with the invasive foodborne pathogen *Listeria monocytogenes*. This protection was related to bacteriocin production, and mutant of *L. salivarius* UCC118 lacking the bacteriocin gene failed to protect mice against infection (Corr et al., 2007). Some similar results were obtained with human *L. lactis* and *Pediococcus acidilactici* nisin- and pediocin-producing strains that were able to reduce vancomycin-resistant enterococci intestinal colonization in a mouse model (Millette et al., 2008).

Although *L. lactis* UL719 had no significant effect on *C. difficile* in this model of human colon, addition of nisin (in Nisaplin^®^form) at 76 μmol/L induced a significant reduction of *C. difficile*. The observed efficacy of Nisaplin^®^against *C. difficile* could be due to a synergy between nisin and salt present in the commercial product. At lower concentration of nisin (19 μmol/L), we did not show any significant effect on *C. difficile*, its rapid adsorption on the surface of the colonic microbiota or its inactivation due to enzymatic activities (proteolysis mainly) could explain this lack of activity (Dobson et al., 2011). Rea et al. (2011) have reported on the effectiveness of other bacteriocins such as lacticin 3147 and thuricin CD against *C. difficile* in a distal colon model. Lacticin 3147 (270 μmol/L) and thuricin CD (90 μmol/L) affected the viability of *C. difficile* (10^6^ CFU/mL) with a loss of detection after 12 h and three log_10_ reduction after 24 h, respectively (Rea et al., 2011). After respective addition of lacticin 3147 (270 μmol/L) and thuricin CD (90 μmol/L), authors have shown a CFU reduction of 4 log_10_ and 1.2 log_10_, but lacticin at 90 μmol/L had no significant effect on the *C. difficile* viability (Rea et al., 2011). In this study, nisin was as effective as lacticin 3147 and more efficient than thuricin CD with a CFU reduction of 3.23 log_10_ with nisin (76 μmol/L) compared to initial time. Besides, three times addition of vancomycin (90 μmol/L) or metronidazole (90 μmol/L) is required to induce a significant effect on *C. difficile* after 24 h (Rea et al., 2011). A single dose of nisin (76 μmol/L) was as effective as antibiotics traditionally used to treat CDIs.

With increase of failures and recurrences in the treatment of CDIs, development of alternative treatments has become necessary. In recent years, use of probiotic bacteria producing antimicrobial molecules (such as bacteriocins) constitute a promising alternative for prevention and treatment of *C. difficile* related diseases. In the study, we have shown that nisin-producer *L. lactis* UL719 was able to survive and proliferate in the human colon model. Although *L. lactis* UL719 failed to inhibit *C. difficile* in this model, *L. lactis* UL 719 had not affected the microbiota. Others studies aiming to increase competitivity and nisin production will be necessary and could include the addition of prebiotics or carbohydrate which stimulate nisin production. Nisin (Nisaplin^®^) causes some temporary changes in the microbiota but is effective at killing *C. difficile* in the human colon model.

## Conflict of Interest Statement

The authors declare that the research was conducted in the absence of any commercial or financial relationships that could be construed as a potential conflict of interest.
